# Patient and public views about the security and privacy of Electronic Health Records (EHRs) in the UK: results from a mixed methods study

**DOI:** 10.1186/s12911-015-0202-2

**Published:** 2015-10-14

**Authors:** Chrysanthi Papoutsi, Julie E. Reed, Cicely Marston, Ruth Lewis, Azeem Majeed, Derek Bell

**Affiliations:** NIHR CLAHRC Northwest London, Imperial College London, Chelsea & Westminster Hospital NHS Foundation Trust, London, UK; Department of Primary Care Health Sciences, University of Oxford, Oxford, UK; Department of Social and Environmental Health Research, London School of Hygiene and Tropical Medicine, London, UK; Department of Sociology, University of the Pacific, Stockton, CA USA; Department of Primary Care & Public Health, Imperial College London, London, UK

**Keywords:** Health information, Security, Confidentiality, Privacy, Patient views, Electronic health records

## Abstract

**Background:**

Although policy discourses frame integrated Electronic Health Records (EHRs) as essential for contemporary healthcare systems, increased information sharing often raises concerns among patients and the public. This paper examines patient and public views about the security and privacy of EHRs used for health provision, research and policy in the UK.

**Methods:**

Sequential mixed methods study with a cross-sectional survey (in 2011) followed by focus group discussions (in 2012-2013). Survey participants (*N* = 5331) were recruited from primary and secondary care settings in West London (UK). Complete data for 2761 (51.8 %) participants were included in the final analysis for this paper. The survey results were discussed in 13 focus groups with people living with a range of different health conditions, and in 4 mixed focus groups with patients, health professionals and researchers (total *N* = 120). Qualitative data were analysed thematically.

**Results:**

In the survey, 79 % of participants reported that they would worry about the security of their record if this was part of a national EHR system and 71 % thought the National Health Service (NHS) was unable to guarantee EHR safety at the time this work was carried out. Almost half (47 %) responded that EHRs would be less secure compared with the way their health record was held at the time of the survey. Of those who reported being worried about EHR security, many would nevertheless support their development (55 %), while 12 % would not support national EHRs and a sizeable proportion (33 %) were undecided. There were also variations by age, ethnicity and education. In focus group discussions participants weighed up perceived benefits against potential security and privacy threats from wider sharing of information, as well as discussing other perceived risks: commercial exploitation, lack of accountability, data inaccuracies, prejudice and inequalities in health provision.

**Conclusions:**

Patient and public worries about the security risks associated with integrated EHRs highlight the need for intensive public awareness and engagement initiatives, together with the establishment of trustworthy security and privacy mechanisms for health information sharing.

**Electronic supplementary material:**

The online version of this article (doi:10.1186/s12911-015-0202-2) contains supplementary material, which is available to authorized users.

## Background

Information privacy and security issues have long been debated in the context of technological change and electronic databases in healthcare [1–5]. Despite extensive ethical and technical analysis, concerns remain as the effort to make health information accessible to and usable by a wide range of health professionals, researchers and planners often conflicts with notions of patient confidentiality and autonomy [6–8]. In the UK, privacy and security concerns are repeatedly discussed in popular media with numerous reports of patient information being stolen, lost, misplaced or released without authorisation (see [9] for a review). Areas of concern were also highlighted in the 2013 Information Governance Review, which noted that 186 serious data breaches had been reported to the Department of Health between July 2011 and June 2012 in England [10]. The landscape is changing rapidly, with new challenges emerging as re-identification techniques for previously anonymised data develop, and ethical questions raised by the increasing attention to genomic information become more urgent [11–13].

Empirical work on patient and public attitudes to technological implementation and integrated Electronic Health Records (EHRs) for health provision and secondary purposes has to date identified generally positive attitudes towards wider health information sharing and record linkage, with some variation in views between different socio-demographic groups and between individuals with different levels of personal exposure to health services [14–18]. However, information security and privacy assurances, such as strong anonymity and consent mechanisms, continue to play a significant role in sustaining patient trust in record keeping practices [14, 17, 19–28]. Patients may choose to withhold information and delay seeking treatment rather than reveal specific types of health information, especially when they perceive they have little control over its use [29–34]. Even sharing information between health professionals may raise patient concerns, for example in the case of potentially stigmatising conditions [35, 36].

In this study we explore (i) the extent to which patients and the public report being worried about the security of integrated EHRs (as systems including longitudinal clinical information from different sources and used simultaneously for different purposes, such as care provision, research and planning); (ii) how these worries relate to levels of support about the development of EHRs; (iii) what differences exist between groups with different socio-demographic characteristics, health conditions and patterns of interaction with the health service. Findings from a quantitative survey are combined with analysis of qualitative focus group discussions to further explore the rationale behind patient support and concerns about EHRs.

## Methods

This paper presents findings from a sequential mixed methods study using an initial survey questionnaire to elicit high-level patient and public perceptions on different questions about EHRs, which were then examined in more detail in a series of focus group discussions. The two phases of the study are explained in the following sections.

### Phase 1: survey questionnaire

A cross-sectional survey questionnaire was administered to patients and members of the public from a stratified cluster random sample of 8 waiting rooms of General Practice (GP) surgeries and 8 outpatient waiting areas of a teaching National Health Service (NHS) hospital in West London between August and September 2011.

To develop the questionnaire the project team carried out an extensive review of the literature and identified factors that might influence how patients and the public view EHRs (e.g., age, health status, frequency of healthcare visits, educational background, ethnicity). Through multiple iterations this framework provided the foundation to construct the survey questions which were then compared with existing tools measuring patient attitudes on the topic. C*ardiff TELEform* survey software was used to develop the final version of the questionnaire which was then piloted for use with 30 adults (over age 18) from the general population. Pilot participants were selected from nurse patient advocacy groups, patient and public involvement networks, and personal contacts of the research team. Participants varied according to age, gender, education, ethnicity, parents, carers, people with and without long-term health conditions, and people with differing levels of experience with healthcare practice and research. Multiple rounds of piloting and revision of the questionnaire were conducted with the same sample of 30 individuals over a period of three months until all participants responded that they understood each question, accepted the design and layout as a whole, and were able to complete it within ten minutes [37].

Recruitment for the main survey was carried out in hospital waiting areas where the team had the opportunity to approach patients living with a diverse range of health conditions as they waited for their appointments with different specialties: orthopaedics, bariatric, urology, maxillofacial, pain, diabetes, HIV and sexual health, dermatology, eye, antenatal and phlebotomy. Participants filled in the questionnaire on their own without receiving any help or other instructions from the researchers. A random sampling design was used to minimise selection bias, with recruitment carried out on different days and at different times, divided equally between GP surgeries and the hospital. Only individuals over 18 years old who were able to understand the information describing the study in English were eligible to participate. All participants provided informed consent on the first page of the questionnaire.

The front page of the survey defined EHRs as a record that, if created, would store everything about a patient’s health and healthcare visits from birth until death, bringing together under one record all separate paper and electronic files held across different health providers. The questionnaire contained 31 items, including questions on socio-demographic characteristics (birth year, sex, ethnicity, highest level of educational attainment) and health-related information (long-term health conditions and frequency of health visits). Survey questions on views about EHR security were:

1. If your record was part of a national electronic records system, would you worry about the security of your record? (Response options: Yes/No)
2. Do you think the NHS is presently able to make electronic health records secure? (Response options: Yes/No)
3. How do you feel about the security of electronic health records compared to your current health records? (Response options: Less secure/Equally Secure/More secure)

The data analysis further compares views about EHR security, identified through the questions above, with support for the development of EHRs as measured in the same survey (Response options: Yes/No/Undecided) and examined more closely in an earlier publication [15]. A copy of the survey questionnaire and other methodological details on the quantitative phase of the study are published elsewhere [37].

### Survey data analysis

**Fig. 1:**
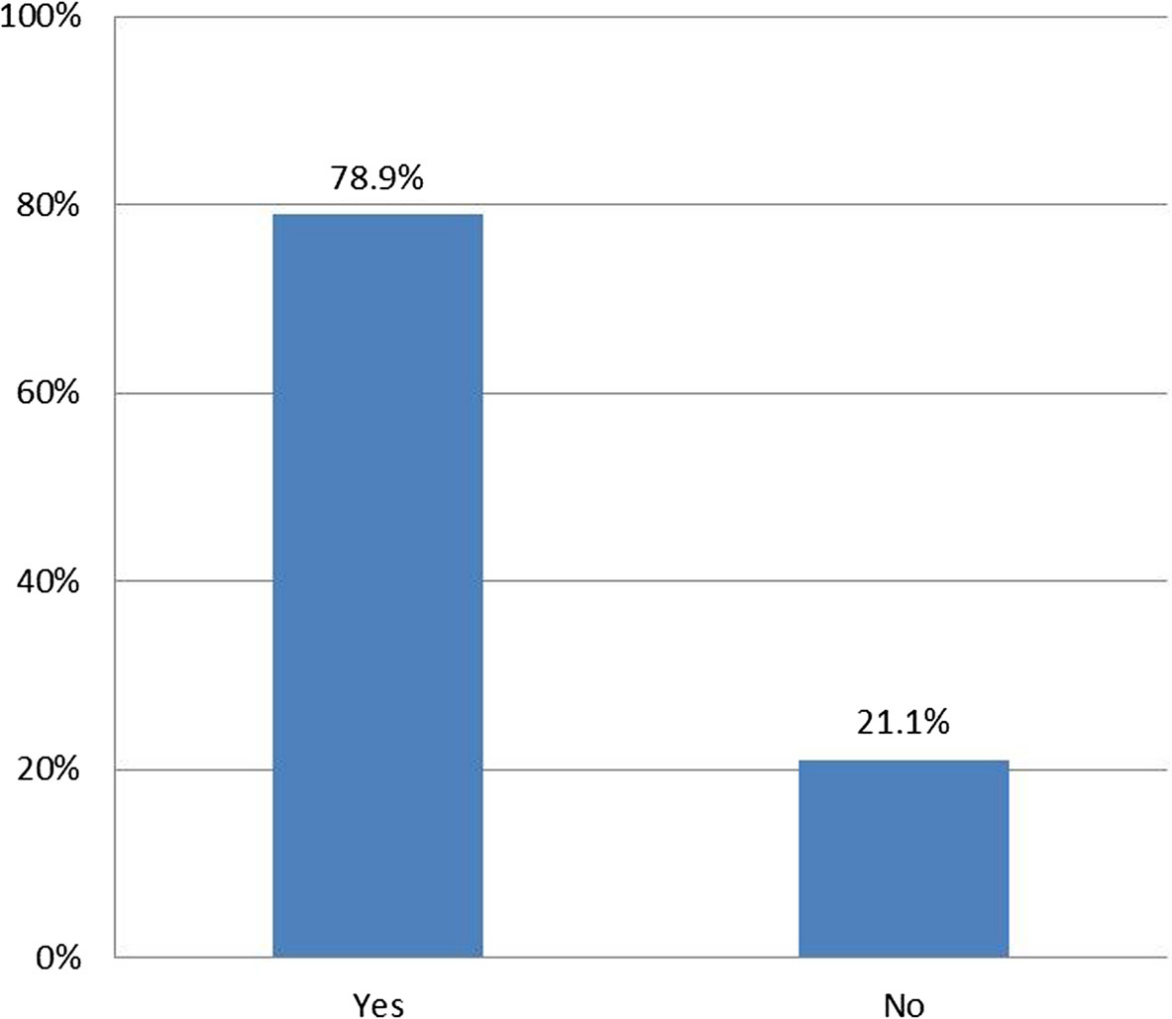
Respondent views on whether they would worry if their record was part of a national EHR system, *N* = 2761

**Fig. 2:**
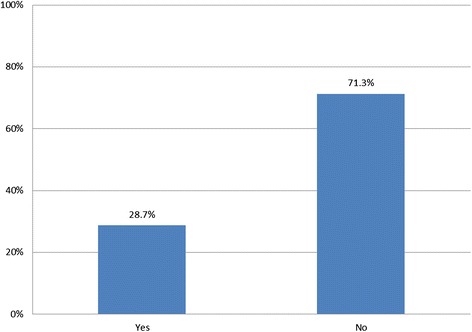
Respondent views on the ability of the NHS to maintain EHR security at the time of the survey, *N* = 2761

**Fig. 3:**
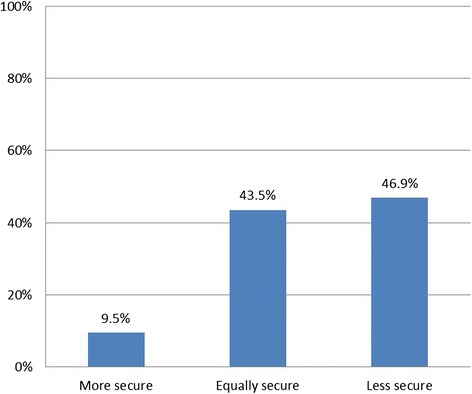
Respondent views about EHR security compared with their own records, *N* = 2761

We conducted univariate analysis to describe our sample (Table 1) and to determine the proportions of people in each of the response options for our outcome variables (Figs. 1, 2 and 3). Using chi-squared tests of statistical significance, we examined perceived security views against overall support for EHRs (Table 2). In logistic regression analysis we assessed participants’ socio-demographic and health-related characteristics in relation to their views about security of national EHRs and their confidence in the ability of the NHS at the time of the survey to maintain EHR security (Table 3). We used multivariate multinomial regression models to examine the relationships between perceptions of EHR security compared with perceptions of security of current records, taking respondent socio-demographic and health-related characteristics into account (Table 4). Regression models only included independent variables significantly associated with the outcome of interest in bivariate analysis (*p* < 0.05).

**Table 1:** Socio-demographic and health-related characteristics of the sample, *N* = 2761

	% (N)
Age category	
18–24	8.1 (225)
25–34 (base)	26.5 (732)
35–44	21.2 (584)
45–54	15.5 (427)
55–64	11.8 (325)
65–74	10.5 (289)
75+	6.5 (179)
Sex	
Female (base)	59.1 (1,633)
Male	40.9 (1,128)
Ethnicity	
White British (base)	56 (1,546)
White Non-British	20.2 (559)
Black/African/Caribbean/British Black	7.4 (205)
Asian/Asian British	8.1 (223)
Mixed/Multiple	8.3 (228)
Educational qualifications	
No academic qualification	4.9 (136)
GCSE	11.2 (310)
A-Levels	10.2 (282)
Vocational qualification	12.4 (342)
Degree	36.4 (1,006)
Higher Degree (base)	24.8 (685)
Clinic type	
GP clinic (base)	33.6 (927)
Outpatient	66.4 (1,834)
Frequency of healthcare use in past 6 months	
0 to 2 (base)	36.4 (1,004)
3 to 5	34.7 (957)
6 to 9	15.9 (439)
10 plus	13.1 (361)
Long term conditions	
No conditions (base)	34.8 (961)
At least one condition	65.2 (1,800)
Total	100 (2,761)

**Table 2:** Relationships between EHR security perceptions and overall support for national EHRs, *N* = 2761

	Overall, are you in favour of the development of a national electronic health records system?			
	Yes	No	Undecided	Total
	% (N)	% (N)	% (N)	% (N)
Worried about security if record was part of national EHR system				
No	86.1 (501)	2.7 (16)	11.2 (65)	100 (582)
Yes	55 (1,199)	12.3 (269)	32.6 (711)	100 (2,179)
Pearson chi2(2) = 188.2000 *p* = 0.000				
Perceptions about NHS ability to safeguard EHRs at the time				
No	53.5 (1,053)	13 (255)	33.5 (660)	100 (1,968)
Yes	81.6 (647)	3.8 (30)	14.6 (116)	100 (793)
Pearson chi2(2) = 190.3913 *p* = 0.000				
Perceived EHR security compared with current records				
Less secure	46.5 (603)	17.4 (226)	36 (467)	100 (1,296)
Equally secure	72.7 (874)	4 (48)	23.3 (280)	100 (1,202)
More secure	84.8 (223)	4.2 (11)	11 (29)	100 (263)
Pearson chi2(4) = 280.8978 *p* = 0.000

**Table 3:** Logistic regression models: perceptions about national EHR security and the ability of the NHS to safeguard EHRs at the time, *N* = 2761

	Worried about security if record was part of national EHR system (base: not worried)			Perceptions about NHS ability to safeguard EHRs at the time (base: unable to safeguard EHRs)		
	RR	95 % CI	p	RR	95 % CI	p
Age category (base: 25–34)						
18–24	1.12	[0.76,1.64]	0.58	0.97	[0.74,1.27]	0.83
35–44	2.15	[1.74,2.66]	0.00	0.60	[0.49,0.74]	0.00
45–54	2.45	[1.75,3.43]	0.00	0.71	[0.56,0.89]	0.00
55–64	1.98	[1.49,2.63]	0.00	0.41	[0.30,0.55]	0.00
65–74	1.10	[0.75,1.63]	0.62	0.62	[0.43,0.90]	0.01
75+	1.18	[0.82,1.69]	0.36	0.46	[0.27,0.79]	0.00
Ethnicity (base: White British)						
White Non-British	0.74	[0.44,1.25]	0.26	0.63	[0.49,0.79]	0.00
Black British	0.74	[0.45,1.22]	0.24	1.15	[0.84,1.56]	0.38
Asian British	0.57	[0.35,0.93]	0.02	1.52	[0.95,2.43]	0.08
Mixed/Multiple/Other	1.10	[0.54,2.21]	0.80	1.21	[0.86,1.70]	0.28
Education (base: higher degree)						
None	0.44	[0.30,0.65]	0.00	2.60	[2.04,3.32]	0.00
GCSE	0.68	[0.42,1.09]	0.11	1.62	[1.13,2.34]	0.01
A-Level	0.71	[0.51,1.00]	0.05	1.43	[1.06,1.94]	0.02
Vocational	0.87	[0.54,1.43]	0.59	1.53	[1.06,2.21]	0.02
Degree	0.86	[0.68,1.08]	0.20	1.07	[0.88,1.30]	0.53

The first logistic regression model compares those who would worry about the security of their record if this were part of a national EHR with those who would not worry. The second logistic regression model compares those who think the NHS was able to make electronic health records secure at the time of the survey with those who do not think the NHS was able to do so. *P*-values are adjusted for clustering by sampling site

**Table 4:** Multinomial regression model on perceptions of EHR security compared with current records, *N* = 2761

	Perceptions of EHR security compared with current records (base: less secure)					
	Equally secure			More secure		
	RR	ci95	p	RR	ci95	p
Age category (base: 25–34)						
18–24	1.24	[0.90,1.69]	0.19	1.28	[0.77,2.13]	0.35
35–44	0.67	[0.54,0.82]	0.00	0.68	[0.53,0.87]	0.00
45–54	0.56	[0.44,0.71]	0.00	0.52	[0.30,0.90]	0.02
55–64	0.52	[0.39,0.69]	0.00	0.57	[0.39,0.83]	0.00
65–74	0.65	[0.46,0.92]	0.02	0.60	[0.37,0.95]	0.03
75+	0.63	[0.46,0.85]	0.00	0.63	[0.39,1.01]	0.06
Ethnicity (base: White British)						
White Non-British	1.25	[1.02,1.53]	0.03	1.45	[1.05,2.00]	0.03
Black British	0.74	[0.54,1.00]	0.05	1.48	[0.99,2.20]	0.05
Asian British	1.26	[1.04,1.53]	0.02	2.28	[1.52,3.44]	0.00
Mixed/Multiple/Other	1.13	[0.88,1.45]	0.34	1.91	[1.17,3.11]	0.01
Education (base: higher degree)						
None	1.55	[0.96,2.51]	0.07	3.05	[1.51,6.17]	0.00
GCSE	1.58	[1.05,2.38]	0.03	2.32	[1.48,3.65]	0.00
A-Level	1.26	[0.97,1.64]	0.08	1.50	[0.87,2.58]	0.14
Vocational	1.10	[0.75,1.60]	0.62	1.31	[0.85,2.03]	0.22
Degree	1.01	[0.78,1.32]	0.93	1.27	[0.96,1.67]	0.09

Multinomial regression model comparing those who feel that integrated electronic health records are as secure as or more secure than their current health records, with those who report that integrated records would be less secure (base). *P*-values are adjusted for clustering by sampling site

Participants who provided incomplete responses for one or more variables were excluded from the final analysis (48.2 %). To examine potential differences between respondents who were included in the analysis sample with those who were excluded, we performed missing data analysis using logistic regression models (results included in Additional file 1). *P*-values and 95 % confidence intervals were adjusted for clustering by sampling site in all cases. Data analysis was carried out using STATA IC 9.0.

### Phase 2: qualitative study

To explore the results of the survey in more detail, we carried out 17 qualitative focus group discussions with a total of 114 patients living with different conditions and carers (September 2012 – April 2013). Four of the focus groups also included health professionals, health researchers and NHS managers to further understand any differences in opinion between professionals with experience using EHRs and members of the public. Individuals interested in the study who did not wish to join a group discussion were interviewed separately (6 participants).

Our sampling methodology was driven by the survey findings and aimed to capture the widest range of perspectives possible. Recruitment was carried out through patient organisations and support groups, West London GP practices and hospitals, and carers’ centres. Condition-specific focus groups and interviews covered a wide range of areas including people living with diabetes, asthma and allergies, thyroid disorders, rheumatoid arthritis, heart conditions, sickle cell disorders and HIV. We also organised focus groups to specifically examine the views of older and younger people, those identifying as black minority ethnic groups, and carers (one focus group per category).

Focus groups lasted between 60–110 min and interviews between 30–45 min. Two researchers experienced in qualitative research supported the discussions, with one primarily responsible for facilitation and the other for taking detailed notes. An indicative focus group guide was used including questions on participants’ experiences with information sharing between different NHS providers, their hopes and concerns around integrated systems used for healthcare, research, and planning purposes, as well as their thoughts on the best ways to involve patients and members of the public in decision-making about the future of EHRs. Additional materials in the form of comic illustrations were also used to increase engagement and trigger discussion around the questions in the topic guide. Participants received a £30 gift voucher to cover travel expenses and to compensate them for their time participating in the discussions.

All interactions were recorded and transcribed verbatim with written consent. We offered participants the option to view their transcripts before the analysis but did not receive any requests. Two researchers carried out thematic analysis using a pre-defined coding framework which they refined through iterative rounds of deductive (codes identified in advance) and inductive (codes emerging from the data) coding. The researchers then compared their findings and discussed differences in their coding with the wider team to enhance understanding and identify nuances in meaning. Preliminary findings were presented to and discussed with a sub-group of participants (*n* = 24) who attended 3 additional workshops organised in June 2013 with the aim of evaluating information materials on EHRs (leaflets and online resources) and developing a video charting the project journey (https://www.youtube.com/watch?v=Uj4kXhBgqe4&feature=youtu.be).

### Research ethics

The study was approved by the London Dulwich Research Ethics Committee (Ref. No. 10/H0808/96). All participants were provided with information sheets, either on the front page of the survey or as a separate document, and gave written consent to participate.

## Results

### Quantitative results

#### Sample characteristics

The overall response rate for the survey was 85.5 %, (*N* = 5331), but fewer participants (*N* = 2761, i.e., 51.8 % of respondents) provided complete data for all variables included in the analysis for this paper (responses with missing data have been excluded – see Additional file 1). Table 1 describes our sample by birth year, sex, ethnicity, educational qualifications, recruitment site, long-term conditions and frequency of healthcare visits. Almost half of the respondents (47.7 %) belonged to relatively young age groups between 25–34 and 35–44 years old, many were female (59.1 %) and more than half self-identified as White British (56 %). Around two thirds of participants reported having degrees or higher degrees (61.2 %), and 66.4 % were recruited from outpatient clinics for the survey. In terms of health-related characteristics, the sample consisted of a large proportion of people with at least one long-term health condition (65.2 %) and a moderate number of visits to health services: 36.4 % of participants had visited health services 0–2 times in the 6 months prior to the survey and 34.7 % had made 3–5 visits in the same period.

#### Respondent views about security

Overall, 78.9 % of respondents stated that they would worry about the security of their health record if it were part of a national electronic records system (Fig. 1). Similarly, 71.3 % voiced doubts about the ability of the NHS to guarantee the security of EHRs at the time the survey was carried out (Fig. 2). Almost half (46.9 %) of respondents said that detailed, integrated EHRs would be less secure than the way they assumed their health records were held at the time of the survey, 43.5 % said security risks would be equal, and 9.5 % that security would be increased (Fig. 3).

#### Relationship between security perceptions and overall support for EHRs

Previously published results drawing on a preliminary analysis from the same study showed moderately high levels of support for the development of national EHRs used simultaneously for healthcare provision, planning and policy, and health research: 62.5 % of participants reported overall support, 27.9 % reported being undecided and 9.6% said they would not support a national EHR system used for multiple purposes [15]. Higher levels of support were reported for specific uses of EHRs. Most participants were in favour of EHRs specifically for personal healthcare provision (89.7 %), for health services policy and planning (79.5 %), or for research (81.4 %), although 59.7 % and 67.1 % of respondents would prefer their personal identifiers to be removed for health policy and research respectively [15].

To further understand whether those stating that they would be worried about national EHRs also report being in favour or against their development, we carried out analysis showing the relationships between the different variables (Table 2). Of those who said they would worry if their records were part of a national EHR system, 55 % nevertheless reported that they would support the development of this system, 32.6 % were undecided in their views, while 12.3 % would not be in favour of national EHRs. There was a similar pattern between those who thought that the NHS would be unable to guarantee the security of EHRs at the time of the survey: 53.5 % reported support for the development of national EHRs, 33.5 % were undecided and 13 % would not support the system. Of those who thought EHRs would be less secure compared with current records, 46.5 % said that they would support the development of national EHRs, 17.4 % said they would not support them, and 36 % reported being undecided. People who did not report being worried about national EHR security were not necessarily fully supportive of EHR development: 11.2 % said they were undecided and 2.7 % that they were not in favour of the development of this system. We explored these findings in greater detail in focus group discussions (presented in a separate section).

### Associations between security perceptions and socio-demographic and health-related characteristics

We used logistic regression models to identify associations between socio-demographic and other health-related characteristics of the sample, in relation to views about EHR security and what participants thought about the ability of the NHS to safeguard EHRs when the survey was conducted (Table 3). Respondents between 35 and 64 years old were more likely to report that they would be worried about the security of their records as part of an integrated EHR system (OR = 1.98 to 2.45, *p* < 0.05) than the base group consisting of individuals 25–34 years old (for age categories over 64 years old differences from the base group were not statistically significant). Respondents over 35 years old were also more likely to report less confidence in the ability of the NHS to safeguard EHRs when the survey was conducted (OR = 0.27 to 0.71, *p* < 0.05), than participants aged 25–34. Individuals with no academic qualifications were less likely to say that they would worry about security if their record were part of a national EHR (RR = 0.44, *p* < 0.05), compared with participants with higher degrees. Reported confidence in the NHS to make EHRs secure at the time of the survey was similarly inversely related to educational levels.

After adjusting for all variables in multivariate multinomial models, we identified further age, ethnicity and education differences in security perceptions between integrated EHRs and established systems (Table 4). In comparison with the base group aged 25–34, participants over 35 years of age were less likely to report that integrated EHRs would be equally (RR = 0.52 to 0.67, *p* < 0.05) or more (RR = 0.52 to 0.68, *p* < 0.05) secure compared with the system they thought their health providers used for patient record management at the time the survey was completed. Individuals who self-identified as White non-British, Asian/Asian British and Black/African/Caribbean/British Black were more likely to respond that EHRs would be as secure as (OR = 1.25 and 1.26 respectively, *p* < 0.05) or more secure than (OR = 1.45 and 2.28 respectively, *p* < 0.05) the existing system, compared with White British groups. Those identifying as Black/African/Caribbean/British Black were also more likely to say that EHRs would be more secure (RR = 1.48, *p* = 0.05) than those self-reporting as White British. Participants with no educational qualifications or holding General Certificates of Secondary Education (GCSEs) were more likely than those with higher degrees to suggest that EHRs would be more secure (RR = 3.05, *p* < 0.05). No other associations between security perceptions, socio-demographic and health related variables were statistically significant.

### Qualitative results

Drawing on the results of the quantitative survey, focus group discussions further explored patient and public views about the security of cradle-to-grave integrated EHRs and the various rationales underlying readiness to support their development.

### Debating benefits for patient care against perceived EHR security risks

Most participants expected EHRs to improve patient care and treatment, from short-term emergencies to long-term multifaceted management of chronic conditions. In particular, they said that wider information-sharing between health professionals could provide the potential for faster diagnosis, more targeted interventions and ‘linked up’ care for patients with complex needs, among other benefits. Not having to repeat medical histories could also enable more dignified care, some said, especially for frail or vulnerable patients.

*But also I think, if the electronic health records would help so that my daughter doesn’t have to have assessment after assessment after assessment, maybe have one assessment and that’s shared between everyone, because she has to constantly have all these assessments, and it’s depressing to have to keep talking about what you can’t do all the time.* (FG10)

Rarely did participants express full support for the development of EHRs without adding any caveats. More often people engaged in a negotiation process where they weighed up perceived benefits from using integrated records against concerns about EHR security and other risks. Hacking and identity theft were frequently mentioned as being of concern, alongside unauthorised access. People said they were particularly worried about insurance companies, employers and ‘*people outside the NHS*’ having access to their records, as the more the information was shared, the more difficult it would be to control:

- *My concern is exactly that: who has access to my files and how can we make sure that only those I want to have access would have access? […]*
- *A record could just be available within the health profession to begin with.*
- *But is [this where] the cancer begins, is [this where] we say, yes, that’s fine [to share with other health professionals] and then it gets taken out of hand five years later?*
- *Now it may be [that we] need to concede that somewhere along the line things may get contaminated. And there might be some exposure [of health information to other occupational groups].* (FG12)

Focus group participants preferred different access levels for different occupational groups, with certain professionals being permitted access to full medical records (e.g., general practitioners) as opposed to a more restricted or limited version being accessible to other professionals who were not as involved with their care. However, they also recognised that there might be legitimate reasons for different staff members to require access to medical records. Some said they would feel uncomfortable with health professionals having access to their information beyond what would be strictly necessary for the situation at hand, for example, instead of pharmacists only being able to see what medication has been prescribed, also being able to access the reasons for the prescription. Others suggested that pharmacists could act as a type of ‘*safety net’* to correct mistakes if they had access to more information. Participants often talked about how particular sensitive details warranted more security measures, although this raised questions about how to ensure that measures are not routinely circumvented, while still being possible to override in emergencies. Discussions also developed to encompass a recognition that ‘*no system is failsafe’*.

*And even if you put in security levels, it’s very difficult because you could justifiably say that most of those 12 [occupational groups], it’s good they should have access to your records. But I take the attitude, and I’ve been in IT, you can have all the security systems you’ve got, but if somebody wants to break into them they’ll break into them.* (FG5)

Participants from socially disadvantaged or ethnic minority backgrounds specifically expressed worries about how information included in patient notes might unduly influence subsequent consultations in different settings. Beyond information seen as potentially stigmatising (e.g., in relation to sexual or mental health) participants said they worried that health professionals might also make character judgments, such as labelling a patient as ‘*hypochondriac’* or as having social problems, and that this could lead to difficulties accessing appropriate care.

*I know it could lead to negative labelling, definitely. And it just comes down to the human level, with the nurse, the GP dealing with patients, how it will affect their treatment of people, I’m sure it will have an influence on that. There will be someone down the line that will react negatively, there’s no doubt about it.* (FG7)

Other participants had found errors in their medical records and worried that if incorrect information was more widely shared it might have consequences on further diagnoses or treatment decisions.

*I now habitually collect a record of everything. If I have blood tests or anything, I will say to the GP I would like a copy of the records […] there’s an awful lot of stuff on my records that isn’t accurate […] and if people aren’t properly informed then they may not be making the right decisions.* (FG2)

### Debating the value of EHRs for research and planning

Most participants said they were happy for large datasets to be shared with researchers and policy-makers if this would lead to better understanding of causes of disease, the development of more effective treatments, and better resource allocation, particularly if this could be of direct benefit to themselves or to future generations. In general, participants said they wanted to be informed if details from their own medical records were shared for purposes beyond their clinical care. Many wanted to know how and why their information might be shared, and who would benefit from this, especially in relation to information being used for research and planning purposes.

*[If they] explain to me that the database is not only for medical purposes but would also get us access to more medical [services] in terms of the way the commissioning is taking place, then yes, you are making a good case to get me on the database, but if you are saying that, oh, I should just provide my [information] what’s this all this research going on for?* (FG13)

Although focus group participants debated the value of anonymisation, there was little consensus on what would qualify as identifying information, or how anonymisation could be achieved effectively. Many wondered whether the information from health records would be reliable and accurate enough to be used for other purposes such as research and planning – ‘*rubbish in, rubbish out’* was one phrase used to convey concern for data quality. However, people living with sickle cell disorders discussed how EHRs would allow the collection of population-level data on conditions where patient populations are smaller or more difficult to locate, which they thought was not done adequately at the time, leading to decision-making bias and inequalities.

*If they want to know how many sickle cell patients is out there, they don’t know it right now, but if they had that database they’ll be able to go there and get more information.* (FG13)

### Ownership and accountability

Although participants highly valued the role of the English healthcare system in terms of care provision, many were sceptical when discussing their views on the ability of the NHS to safeguard medical information and manage large technological projects.

*I just have very little faith in the way that the NHS handles databases. I don’t think it’s got a very good record. […] I know how bad some of the IT systems have been. I’ve had to work with them myself, and we just hope the National Health Service will get this one right. They’ve got a few wrong in the past […]* (FG3)

A previous project introducing integrated electronic systems in the NHS (National Programme for IT) had received negative publicity in the years before the focus groups took place and was eventually scaled down to a much narrower scope than initially envisaged. Many participants raised this in their comments, along with other media reports discussing data breaches in the NHS and other government bodies.

- *Always thought that [the NHS] would mess it up.*
- *I’ve heard a bit about it, that they done it, but they paid lots of money.*
- *Yeah but then what they do is that they leave it on a train, don’t they usually.* (FG11)

Some said that they could not fully trust the NHS, which they characterised as ‘*big and bureaucratic’*: the size and bureaucracy being something they linked with lack of ability to protect sensitive personal and health information. This was particularly the case when they had worked in the NHS themselves.

- *[…] there’s less attention to detail, people are careless, they’re poorly trained and nobody wants to be accountable. […]*
- *And the other thing about the NHS, because I also worked in the NHS as a temporary administrator, people are incredibly badly paid and demotivated.*
- *That’s what I was going to say, temping, they get lots of temps.* (FG9)

Others, however, thought that when information is held by government-controlled bodies, such as the NHS, accountability would be easier to achieve. This discussion led on to many participants expressing concern about the increasing privatisation of the NHS and the impact this would have on information sharing with private companies, where, they said, it would be more difficult to hold people accountable for security breaches.

*But what I’m also trying to communicate is even though your information are [sic] within the government system, you can have an employee who might be paid by [the] private sector.* (FG13)

For many participants commercial exploitation of health information was seen as a detrimental outcome of sharing information with private organisations. Pharmaceutical companies, for example, were not viewed as equal partners in the healthcare system. Although participants commented on their importance for advances in medication and treatment, they also feared that medical information would increasingly be used for purposes that would provide most profit, as opposed to improving the wellbeing of the whole population.

*Transparency is what we all want to talk about. We’re not terribly worried about [the development of EHRs], but don’t use it for profit, just use it for research and so our carers and our family can deal with [health] matters.* (FG3)

In weighing up benefits and risks from increased information sharing, some of the discussions concluded that there would only be a small probability that privacy risks would be realised, and that risks would generally carry significant consequences for specific individuals rather than the majority of the patient population. As long as risks are ‘*controlled’*, some conceded, overall benefits would outweigh any security concerns. However, many participants seemed to remain undecided in their views or said that framing the relationship between benefits and risks in terms of balance might miss dimensions of the problem they would be concerned about, such as aspects of the context that would influence where the balance lies under different circumstances. Many expressed the need to find out more details about how EHRs might look like in practice and how data would be used for different purposes (e.g., administrative, clinical, research, policy) before being able to decide how their preferences may vary.

## Discussion

Among the survey respondents 79 % reported that they would worry about the security of their record if this were part of a national EHR system, with 71 % indicating that in their opinion the NHS was unable at the time of the survey to make EHRs secure as part of a cradle-to-grave system, used simultaneously for purposes of health provision, research and policy. Almost half of the respondents (47 %) felt that EHRs would be less secure than the way their records were kept when the survey was conducted. Of those who reported being worried about the security of their record, many stated they would nevertheless be in favour of the development of national EHRs (55 %), while only 12 % would not support national EHRs. The rest of participants reported being undecided (33 %) which could be due to lack of awareness on the topic or difficulty contextualising the question in their own specific circumstances.

Survey findings were explored in more detailed focus groups discussions in which participants weighed up the potential advantages and disadvantages of implementing EHRs and negotiated their concerns around: unauthorised access, commercial exploitation, lack of accountability, data inaccuracies, prejudice and inequalities in health provision. Focus groups debated the role of different health professionals in the information ecology and considered how appropriate information sharing might be organised, highlighting the importance of providing different access levels to different professional groups and informing patients about how their information is used. Participants also expressed doubts about whether absolute security and privacy can be fully guaranteed within the health service and many took the view that overall benefits would outweigh security concerns for the majority of the population.

Previously published studies describe levels of patient and public concern similar to those identified in this paper. For instance, in 2007 a large Canadian survey showed that almost two thirds of respondents reported being worried about the privacy of their medical information [38]. Follow-up research in 2012 found little change in safety and security perceptions of the Canadian public [39]. In the US, a nationally representative study with 3959 participants showed that two-thirds of adults would be concerned about the security of medical information transferred between healthcare professionals electronically or by fax [34]. Although concerns about personal privacy and loss of control have also been expressed in a number of previous UK studies, these primarily focused on the use of medical records for healthcare provision or for research rather than encompassing multiple purposes including healthcare planning [14, 20].

This study extends previous research by providing a measure of the balance between support for the development of national EHRs and views about EHR security. The results suggest that more than half of those who reported being worried about the security of their record in a national EHR system and the ability of the NHS to safeguard integrated EHRs would nevertheless support their development. In the Canadian study mentioned above, respondents also thought that avenues for meaningful use of health technologies should still be explored despite privacy concerns [38]. A US study looking at participation in biobank research highlighted significant privacy concerns which did not correlate with resistance to participation in biomedical studies [40]. Privacy concerns were also expressed in an Irish mixed-methods study of public attitudes towards using health information from GP records for research, yet 89.5 % of respondents suggested that they would still agree to their GP sharing details with researchers [41]. The latter study reflects how patient trust in specific health professionals (e.g., general practitioners) might contribute to willingness to share information for purposes beyond clinical care.

Our survey results emphasise the importance of recognising differences between socio-demographic groups in the UK to better understand and respond to patient views and expectations. In this study people who self-identified as belonging to an ethnic group other than White British were more likely to suggest that EHRs would offer better security than existing record keeping systems. An equally large-scale study on the use of personal, identifiable information by the National Cancer Registry for public health research and surveillance, showed that people from non-white British ethnic groups were more concerned about privacy breaches than White British participants [17]. These findings deserve further exploration to understand differences in patient views and preferences in more detail and adapt policies accordingly.

Opinion differences identified in this study between those with lower education levels who were more likely to have confidence in the ability of the NHS to guarantee EHR security than those with degrees diverge from the Canadian 2007 study, where those with higher educational attainment had fewer concerns about privacy and the protection of personal information [38]. We hypothesise that the idiosyncrasies of the Canadian and British regulatory environments might explain this distinction, together with the differing history of reported data breaches and penalties imposed in the two countries, although further work is needed to understand differences between countries and what implications this might have in practice.

In our study, 35–64 year olds were more likely to report being worried about the security of their record in an EHR system and less confident in the ability of the NHS to safeguard EHRs at the time, compared with 25–34 year olds. Similar differences were noted in a US nationwide survey conducted in 2011, where people under 40 were less sceptical about potential privacy implications of EHRs [42]. Differences in confidence levels may be explained by a number of factors such as different levels of concern about disclosure of health information to employers, or existence of a longer medical history with potentially more conditions that people might not want to share, particularly in the absence of any meaningful penalties for security breaches.

### Situated privacy preferences and conditional sharing

As reflected in our focus group discussions, privacy is rarely static and one-dimensional, but takes a number of contextual, situated and relative meanings [43–46]. Patient willingness to share health information depends on a number of different parameters, including the extent to which patients trust those who receive their information and the perceived sensitivity of the information being shared [47]. Previous research also suggests that people would be more cautious about sharing identifiable personal information with other groups beyond clinical staff [22]. Other studies similarly find that some patients would refrain from sharing certain types of information for care improvement or public health purposes [18]. When thinking about disclosing information in medical settings, individuals seem to perform risk-benefit calculations [14], in what has been termed ‘privacy calculus’ in studies outside the healthcare arena, whereby individuals weigh personal gain against potential harm from information disclosure [48–50]. Since support for integrated EHRs and wider health information sharing is not unconditional, anonymity and consent remain important mechanisms in sustaining public trust, along with stronger controls and effective communication mechanisms [20, 23, 41, 47]. Persisting concerns dependent on changes to the context, personal circumstances and preferences, indicate that ‘granular’ privacy control over which health information should be shared with whom remains an important issue that deserves further research and development in practice, as has been discussed in previous work [51].

### Policy and practice implications

Integrated EHRs containing longitudinal information shared for a number of different purposes, including health provision, research and planning, are viewed by governments, policy-makers and health authorities as an important tool for improving health services, patient safety and other clinical outcomes, as well as for research [52]. Recent policy initiatives in the UK propose wider sharing of health information within the healthcare system and external entities such as pharmaceutical companies and increased use of the NHS number as a unique patient identifier for all medical encounters [52, 53]. These plans have already raised concerns among patient communities, health professionals and advocacy groups about their implications for information privacy and medical confidentiality, with the most recent controversy concerning the ‘care.data’ GP data extraction initiative which aims to draw on data collected in primary care for research and health planning [54–56].

Our survey and focus group findings show that patients and the public would worry about the security of EHRs and had similar concerns regarding the ability of the health service to safeguard information. Their views were often accompanied by uncertainty around what EHRs might look like and what wider information sharing with researchers and policy-makers might mean in practice. In light of recent work suggesting that people prefer to withhold information from medical professionals in the presence of security concerns [34], it is essential to identify ways to ensure both meaningful use of health information and trustworthy sharing practices that safeguard patient privacy. Priorities for further exploration and practical application might include: privacy by design principles that incorporate privacy protection in the design stage, rather than viewing it as an add-on requirement [57], privacy enhancing techniques [58], differential mechanisms that enhance de-identification in database searches [59, 60], dynamic context-aware policies [61], purpose-based policies [62] and notification of privacy breaches to data subjects [63]. Patient control and choice could also be increased through the use of dynamic informed consent and revocation options, formally allowing nuanced preference management as part of EHR systems [64]. This would support patients in contextually negotiating the advantages and disadvantages of sharing information in different circumstances.

Public education about EHRs and meaningful engagement with record keeping could enhance patient input and address concerns by establishing systems that respond to people’s expectations. Age, ethnicity and education differences in security perceptions should be taken in account to provide clear messaging and to effectively explain how any security measures would prevent large-scale attacks, how access to records would be monitored, but also what privacy risks cannot be adequately addressed, such as the potential for patient re-identification. Improved understanding of the reasons why people might feel undecided in relation to electronic records (e.g., because of security concerns or lack of clarity around what EHRs mean or the extent to which private organisations have access) would allow a more targeted approach to address sources of uncertainty.

### Limitations and future research

As part of a mixed methods design, this paper presents one of the few large-scale surveys exploring patient and public perceptions about EHR security, drawing on a comprehensive view of EHRs used for multiple purposes to examine these concerns in the UK. While recruitment for the survey was primarily carried out in West London which might have affected the responses received, we took several steps to minimise selection bias, including using a number of different recruitment points (16 in total), and approaching people at different dates and times of the day. The analysis adjusted for confounding factors, although there might be variables that we have been unable to account for. Qualitative discussions also included different socio-demographic groups to ensure we captured a wide range of perspectives.

Inviting responses on privacy preferences without raising privacy awareness can be challenging, as has been well documented in the literature on participant priming [65, 66]. People who agreed to participate in this research might not be as privacy sensitive as individuals who refrained from taking part, which may have affected the representativeness of responses. The survey referred to general perceptions about EHR security rather than examining specific concerns, which were then explored in more depth as part of the focus groups. In addition, survey participants might have interpreted the questionnaire differently, especially with regard to items about security or privacy which might take multiple contextual meanings. As the survey asked whether participants thought ‘the NHS is *presently* able to make electronic health records secure’, views may have changed since the survey was conducted, although findings from focus groups discussions indicate persisting security worries based around personal experiences with information technologies in the health service.

The definition of EHRs provided to survey participants conveyed UK policy initiatives that were in place when the survey was designed, but have since been reviewed to include less ambitious, smaller-scale technological solutions. The notion of national EHRs used for multiple purposes might have affected participant responses in a way that requires survey findings to be interpreted for the current policy environment. This has been addressed in the group discussions presented here where the focus shifted from national EHRs to a consideration of smaller integrated solutions bringing together different parts of the health service. Future research could focus on the views and perceptions of privacy sensitive groups (for one example see [16]), also expanding on the nuanced nature of privacy needs by considering specific examples of EHR implementation and targeted areas of concern to address this multi-dimensional and complex area of study.

## Conclusions

This work suggests that patients and members of the public remain worried about certain aspects of EHR security, such as the extent of information sharing, governance and accountability risks, the potential for unauthorised access and prejudice, errors and inaccuracies, as well as use of health data for profit and exploitation. Our findings have significant implications for information sharing practices, particularly in the context of the roll out of the primary care data extraction service in the UK, and other similar initiatives worldwide. Meaningful public engagement and transparency are necessary to communicate clearly about the aims of integrated EHRs and to negotiate the boundaries between what patients and the public deem acceptable to share, and how health providers or researchers feel health information should be made accessible. This includes recognition of age, ethnicity and education differences in patient and public views about EHR security as identified in this study. Strengthening information-sharing protocols and protection mechanisms to account for the contextual and situated character of privacy preferences and risk-benefit calculations, while allowing for informed patient participation and choice, may help increase confidence in the ability of the health service to manage and share patient information safely.

## Additional file

Additional file 1:
**Missing data analysis.** Logistic regression analysis of missing data, comparing those who were included in the final sample with those who were excluded because of missing data in one or more variables. *P*-values and 95% confidence intervals are adjusted for clustering by sampling site (Total *N* = 5331). (DOCX 16 kb)

## Abbreviations

EHRs: Electronic Health Records
GP: General Practice
NHS: National Health Service
