# Supplementary material for: Patient and public views about the security and privacy of Electronic Health Records (EHRs) in the UK: results from a mixed methods study
Source: BMC Med Inform Decis Mak. 2015 Oct 14;15:86. doi: 10.1186/s12911-015-0202-2 (PMC4607170; doi:10.1186/s12911-015-0202-2)
Supplement: Additional file 1: — Missing data analysis. Logistic regression analysis of missing data, comparing those who were included in the final sample with those who were excluded because of missing data in one or more variables. P-values and 95% confidence intervals are adjusted for clustering by sampling site (Total N = 5331). (DOCX 16 kb) [file 12911_2015_202_MOESM1_ESM.docx]

**Additional files**

**Table 5. Missing data analysis.**

Logistic regression analysis of missing data, comparing those who were included in the final sample with those who were excluded because of missing data in one or more variables. P-values and 95% confidence intervals are adjusted for clustering by sampling site (Total N=5331).

|  | **RR** | **ci95** | **p** | **N missing (%)** |
| --- | --- | --- | --- | --- |
| **Age category (base 25-34)** | | | | |
| 18-24 | 0.95 | [0.73,1.23] | 0.69 |  |
| 35-44 | 0.92 | [0.76,1.10] | 0.34 |  |
| 45-54 | 0.89 | [0.73,1.08] | 0.24 |  |
| 55-64 | 0.97 | [0.79,1.19] | 0.78 |  |
| 65-74 | 1.01 | [0.80,1.28] | 0.91 |  |
| 75+ | 0.84 | [0.67,1.06] | 0.15 | 799 (15.0) |
| **Sex (base female)** | | | | |
| Male | 1.14 | [1.01,1.29] | 0.04 | 611 (11.5) |
| **Ethnicity (base White British)** | | | | |
| White Non-British | 1.11 | [0.91,1.35] | 0.30 |  |
| Black British | 0.68 | [0.54,0.84] | 0.00 |  |
| Asian British | 0.74 | [0.56,0.97] | 0.03 |  |
| Mixed/Multiple/Other | 0.82 | [0.66,1.01] | 0.07 | 1109 (20.8) |
| **Education (base higher degree)** | | | | |
| None | 0.58 | [0.45,0.76] | 0.00 |  |
| GCSE | 0.91 | [0.79,1.07] | 0.25 |  |
| A-Level | 0.79 | [0.66,0.95] | 0.01 |  |
| Vocational | 1.01 | [0.82,1.24] | 0.93 |  |
| Degree | 0.86 | [0.75,0.99] | 0.04 | 833 (15.6) |
| **Clinic type (base GP clinic)** | | | | |
| Outpatient clinic | 0.99 | [0.87,1.13] | 0.92 | 0 (0) |
| **Frequency of healthcare use (base 0-2)** | | | | |
| 3-5 | 1.14 | [1.01,1.29] | 0.04 |  |
| 6-9 | 1.42 | [1.22,1.64] | 0.00 |  |
| 10 plus | 1.29 | [1.05,1.59] | 0.01 | 686 (12.9) |
| **Long term conditions (base no conditions)** | | | | |
| At least one condition | 0.74 | [0.66,0.84] | 0.00 | 1103 (20.7) |
| **Number of different healthcare services visited (base 0-1)** | | | | |
| 2 | 0.82 | [0.74,0.91] | 0.00 |  |
| 3 plus | 1.10 | [0.94,1.29] | 0.23 | 593 (11.1) |
| **Overall support (base yes)** | | | | |
| No | 0.53 | [0.45,0.61] | 0.00 |  |
| Undecided | 0.72 | [0.65,0.79] | 0.00 | 584 (11.0) |
| **Security concerns (base yes)** | | | | |
| No | 1.04 | [0.96,1.13] | 0.33 | 537 (10.1) |
| **Trust the NHS to make EHRs secure (base no)** | | | | |
| Yes | 0.97 | [0.87,1.08] | 0.59 | 764 (14.3) |
| **Security compared to current records (base equally)** | | | | |
| Less secure | 0.93 | [0.81,1.05] | 0.24 |  |
| More secure | 1.00 | [0.86,1.15] | 0.95 | 650 (12.2) |
| **Overall security risks (base low risks)** | | | | |
| Moderate risks | 1.18 | [1.02,1.36] | 0.03 |  |
| High risks | 1.05 | [0.93,1.19] | 0.40 | 567 (10.6) |
